# Telemedicine in ophthalmology during the COVID-19 pandemic

**Published:** 2020-09-01

**Authors:** Asela Abeydeera

**Affiliations:** 1 President: Association of Community Ophthalmologists of Sri Lanka (SLACO).

**Can teleconferencing help in delivering eye care services during pandemics? A volunteer effort in Sri Lanka showed promise, but needs to be developed further in order to have an impact at national level.**

In mid-March 2020, the government of Sri Lanka announced a lockdown. This meant that eye care practitioners had to minimise all non-essential eye care services such as outpatient clinics and hospital admissions. In the absence of regular eye clinics, many patients became desperate to find care and treatment for their eye ailments.

Based on concerns raised by patients during a few telephone calls, we recognised the need for a teleconferencing intervention to deliver the services. We used social media applications such as Facebook and WhatsApp to contact and bring on board the members of the Sri Lankan Association of Community Ophthalmologists (SLACO). Together, we launched a Facebook page (**www.facebook.com/The-Eye-Patient**) to provide information about common eye ailments, and measures to take, so that patients could refer to it during the lockdown.

Our Facebook posts reached thousands of people from different parts of the country who were seeking help. We encouraged them to submit close-up images of the affected eye(s). In a few instances, with the consent of the patients, optometrists in remote areas volunteered to record a short voice/video clip along with the pictures. A member of our team was usually able to speak to the patient over the phone.

A total of 29 patients got in touch with us via the Facebook page. Their complaints included:

- Red-eye with tearing and discharge, resembling viral conjunctivitis
- Lumps on the eyelids: chalazia, cysts of Zeiss, or cysts of Moll
- Ocular trauma: chemical and mechanical injuries to the eye
- Complications after cataract surgery, such as pain, irritation, and reduced vision
- Itching and red eye, suggestive of allergy
- Swollen eyelids with itching, suggestive of severe blepharitis
- Acute, severe pain and red eye, suggestive of corneal ulcer or endophthalmitis.

## How we addressed these complaints

1. We first ruled out the possibility of there being an ophthalmic manifestation of COVID-19. We noted the history and other clinical features.
2. For cases of trauma and acute endophthalmitis, we suggested an urgent referral to a public sector hospital or eye unit.
3. For chalazion, allergies, blepharitis and conjunctivitis, we prescribed eye drops (that were available free of charge whenever possible) after the teleconsultation.
4. For patients with glaucoma, we supplied eye drops.
5. For those who lost or broke spectacles, or needed them for reading, we supplied free spectacles.
6. We provided health advice or reassurance for delayed cataract surgery or the absence of glaucoma treatment.
7. We referred patients who needed refraction and a spectacle prescription to local optometrists.

According to public health experts, the COVID-19 pandemic will last from several months to a few years. This means that physical distancing and restrictions to services will limit access to eye care services.

Although the numbers were low, the ophthalmologists involved in this initiative were satisfied that patients had received appropriate care.

SLACO now plans to extend this initiave nationally, by:

- Launching an eye care helpline (voice calls in three languages).
- Making the public aware through social media, YouTube and mass media.

We hope that the lessons learnt from this intervention can be used to improve access to eye care services in Sri Lanka; for example, by developing a systematic teleophthalmology model. This could include video consultations, with primary health care or primary eye care workers carrying out basic examinations at primary-level health institutions. This could reduce the number of people who need to visit public health care institutions, thereby reducing delays and long waiting times in the future.

**Images submitted via the Sri Lankan Association of Community Ophthalmologists’ Facebook page**

**Figure 1:**
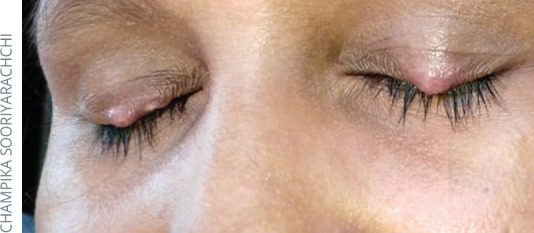
Multiple chalazia

**Figure 2:**
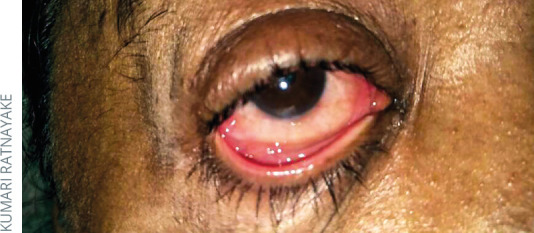
Severe blepharitis

**Figure 3:**
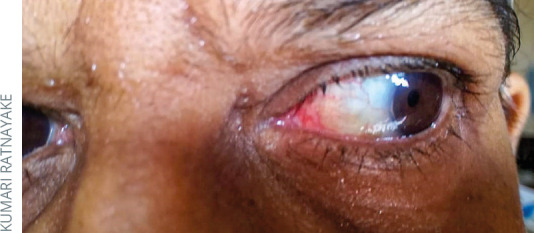
Blepharitis

**Figure 4:**
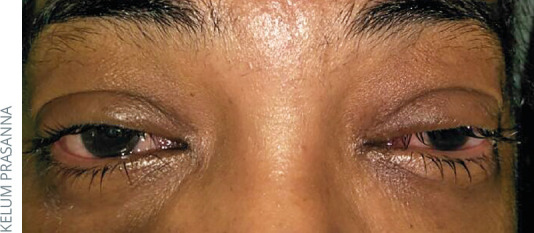
Viral conjunctivitis

**Figure 5:**
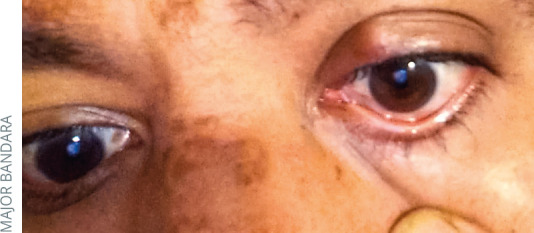
Chalazion and allergic conjunctivitis

